# Insect Pollination Enhances Yield and Physicochemical Quality Traits in Three Jujube Cultivars

**DOI:** 10.3390/insects16121183

**Published:** 2025-11-21

**Authors:** Muhammad Waseem, Danyal Haider Khan, Mudssar Ali, Fawad Zafar Ahmad Khan, Ayesha Hakim, Sami Ullah, Syed Amir Manzoor, Tahir Hussain Awan, Raimondas Mozūraitis

**Affiliations:** 1 Institute of Plant Protection, MNS University of Agriculture, Multan 66000, Pakistan; muhammadwaseem28123@gmail.com (M.W.); danyalhaiderkhan319@gmail.com (D.H.K.); fawad.zafar@mnsuam.edu.pk (F.Z.A.K.); 2 Department of Outreach and Continuing Education, MNS University of Agriculture, Multan 66000, Pakistan; 3 School of Electrical Engineering and Computer Science (SEECS), National University of Sciences and Technology (NUST), Sector H-12, Islamabad 44000, Pakistan; ayesha.hakim@seecs.edu.pk; 4 Department of Horticulture, MNS University of Agriculture, Multan 66000, Pakistan; sami.ullah1@mnsuam.edu.pk; 5 Department of Forestry and Range Management, Faculty of Agricultural Sciences & Technology, Bahauddin Zakariya University (BZU), Multan 66000, Pakistan; amir.kzd@gmail.com; 6 Rice Research Institute, Kala Shah Kaku 54000, Pakistan; tahirawanrri@gmail.com; 7 Laboratory of Chemical and Behavioural Ecology, The State Scientific Research Institute Nature Research Centre, LT-08412 Vilnius, Lithuania; 8 Department of Zoology, Stockholm University, SE-10691 Stockholm, Sweden

**Keywords:** tropical fruit, synchronous protandrous dichogamy, cross-pollination, anemophily, fruit yield

## Abstract

Jujube (*Ziziphus mauritiana* Lam.) is a nutritionally rich tropical fruit which is native to South Asia. In Pakistan, there are 50 varieties of jujube, and approximately 24,000 tons of jujube is harvested annually from a growing area of nearly 5000 hectares. The current study was conducted to investigate whether insect pollination affects fruit yield, and whether it influences the physical and biochemical properties of the fruit. We selected fifty inflorescences from each of the three jujube cultivars (Kheri, Desi, Ayuba) and covered them with muslin cloth to promote self-pollination. The same number was tagged to observe the impact of open pollination. Fruits from both treatments were counted on each panicle, harvested, and then subjected to lab investigation for measuring physical (fruit length, fruit width, fruit weight, and pulp weight) and biochemical (total soluble solids, titratable acidity, vitamin C, and pH) parameters. A diverse range of floral visitors, including bees, flies, wasps, moths, and butterflies, were observed on jujube, with bees being the most abundant functional group. Open pollination increased fruit set (fruits per inflorescence) by 2.3–2.5 times compared to self-pollination. Similar trends were observed in physical parameters: fruit length (1.15 to 1.30 times increase), fruit width (1.15 to 1.21 times), total weight (1.60 to 1.74 times), and pulp weight (1.55 to 1.62 times). Changes in physiological parameters, including pH, vitamin C, titratable acidity, and total soluble solids, were also observed in pollination treatments of all three varieties. Open-pollinated fruit also exhibited greater length and width, but lower firmness. These findings show the vital role of insect-mediated pollination in enhancing both the yield and quality of jujube fruit, with consistent benefits observed across multiple varieties.

## Simple Summary

The current study investigated whether insects facilitate the production of more and higher-quality fruit in jujube trees. In the current study, flowers from three jujube varieties were either left open to enable insect visits or covered with cloth to prevent insect visits to the flowers. The number and quality of fruits were also compared. The results showed that flowers visited by insects produced more than twice as many fruits as those not visited by insects. These fruits had better size, weight, and more pulp. They also had better natural sweetness, acidity, and vitamin content. We observed 11 types of insect pollinators visiting jujube flowers, with honey bees and some fly species being the most abundant insects. This study demonstrates that insects play a crucial role in enabling jujube trees to produce higher-quality fruit. These findings also underscore the importance of protecting insect pollinators to enhance food quality and increase fruit production.

## 1. Introduction

Jujube, *Ziziphus mauritiana* Lam. (Rosales: Rhamnaceae), is a tropical fruit tree native to South Asia [1]. In Pakistan, 50 distinct cultivars of *Z. mauritiana* have been reported to be cultivated on approximately 5000 hectares. The annual fruit production in Pakistan has been estimated at approximately 24,000 tons [2]. This fruit is rich in proteins, carbohydrates, vitamin C, and essential minerals, including iron, zinc, copper, sodium, potassium, calcium, and phosphorus [3]. It provides 1516 to 1575 kJ of energy per 100 g of fruit [4]. In addition to its nutritional value, jujube is widely used in traditional medicine; the leaves, fruits, and seeds are commonly utilised for therapeutic preparations [5,6]. Extracts from the plant have demonstrated anti-tumour and anti-cancer properties [7]. Jujube is well-adapted to arid environments due to its low water requirements, high drought tolerance, and ability to tolerate nutrient-poor soils [8]. The jujube fruit is a drupe that resembles a small apple, with crisp flesh that is the primary edible part and is widely consumed either fresh or processed [9].

For a successful fruit set, the pollination of jujube flowers is essential. The flower of jujube exhibits synchronous protandrous dichogamy, where the male and female stages are distinct and do not overlap, necessitating cross-pollination for successful fruit production [10]. To promote cross-pollination, flowers of different species have evolved specific characteristics to attract pollinators [11]. For example, insect-pollinated plants typically have small, scented, and light-coloured flowers [11], whereas wind-pollinated plants often produce inflorescences with lightweight and diluted pollen [12]. Jujube flowers are characterised as entomophilous due to their small size, greenish-cream colour, and hypanthium-type shape, with a noticeable ammonoid smell during hot and dry days [13]. The pollen is sticky, which limits wind pollination, and flowers have little nectar. Moreover, the swollen floral disc resembles a nectar drop, which also serves to attract insect pollinators through visual cues [14]. Previous studies have reported a diverse assemblage of insect pollinators, including bees, wasps, and flies, visiting jujube flowers [15,16]. Previously, honey bees have been reported as abundant and effective pollinators of jujube, thereby contributing to higher seed viability compared to other pollinator groups [17].

Insect pollination not only enhances fruit set and yield in crops but also influences fruit quality and shelf life [18]. Fruit quality and shelf life depend on various physical and biochemical changes that occur during development, in which pollinators play a significant role [19]. Previous studies have reported that insect-mediated cross-pollination has significantly increased fruit weight in avocado *Persea americana* Mill. (Laurales: Lauraceae) [20], apple *Malus domestica* Borkh. (Rosales: Rosaceae) [21], tomato *Solanum lycopersicum* L. (Solanales: Solanaceae) [22], and phalsa *Grewia asiatica* L. (Malvales: Malvaceae) [23]. Similarly, insect-pollinated fruits have higher total soluble solids in strawberry *Fragaria ananassa* Duchesne (Rosales: Rosaceae) and sweet orange *Citrus sinensis* L. (Sapindales: Rutaceae) [24], lower titratable acidity in lychee *Litchi chinensis* Sonn. (Sapindales: Sapindaceae) [19], and lower pH in cherry *Prunus avium* L. (Rosales: Rosaceae) [25]. In addition, insect pollination has been shown to significantly enhance the bioactive components of medicinal plants, further highlighting the positive impact of insect pollination on the nutritional quality of crops [26,27].

So far, no study has been conducted to evaluate the role of insect pollination in the yield and physicochemical properties of any jujube species. Only a few studies from India have focused solely on the diversity of insect pollinators visiting jujube flowers. The objectives of this study were as follows: (i) to document the abundance and diversity of different insect visitors of the blossoms of three jujube cultivars; (ii) to study the foraging behaviour of the flower visitors of three cultivars to compare the visit duration among the most abundant pollinator groups; and (iii) to assess the impact of two pollination treatments (open vs. caged) on fruit-set probability, fruit yield, and quality.

## 2. Materials and Methods

### 2.1. Study Area

This study was conducted in a *Ziziphus mauritiana* orchard in Multan, Pakistan (30.15° N, 71.44° E) from October to December 2024. The orchard covered an area of 0.7 hectares and was composed of three different cultivars: Kheeri (20 trees), Desi (20 trees), and Ayuba (50 trees). Tree pruning was performed each year during late winter/early spring to remove dead parts and to improve air circulation. Irrigation was provided through an open basin system, where water was applied around the root zone in basins around each tree. Irrigation frequency was adjusted according to the water requirements during the flowering and fruit stages. There was no intercropping in the orchard. Farmyard manure was used as a source of organic matter, and its application was conducted once per year during the spring. Weed removal was performed manually using traditional methods, including hoeing and hand-pulling. A canola field surrounded the orchard to the east and residential buildings to the west. Multan experiences a transition from a hot desert to a hot semi-arid climate [28]. Summers are long and extremely hot, with the mean daily maximum temperature reaching about 41 °C in June, and heatwaves can increase the temperature to 49–51 °C. Winters are short and mild, characterised by night-time low temperatures between 3 and 10 °C, while daytime highs range from 20 to 22 °C. In the winter, dense fog and smog frequently blanket the city from December to January. Rainfall is scarce and highly variable, with most occurring in July and August, averaging 200 mm.

### 2.2. Abundance and Diversity of Flower Visitors of Jujube

Jujube flowers are small, open, and grow in clusters. Pollination is primarily insect mediated. The abundance and diversity of flying insect pollinators were recorded three times daily at 08:00, 12:00, and 16:00 throughout the flowering period, from the first week of October to the second week of December. At each time interval, 15 jujube trees per cultivar were randomly selected to quantify insect pollinator abundance on open inflorescences. The data collection began at the onset of the flowering period, when 10% of the jujube flowers had opened. Timed observations of four minutes per tree were conducted on clear, sunny days, using a stopwatch to record all insect visitors. For each tree, data were collected for one minute on each of the four canopy sides (east, west, north, and south) within a 1 m^2^ area. The insect pollinators were identified using taxonomic keys [29,30].

### 2.3. Visit Duration of Pollinators Among Varieties

The foraging behaviour of the most abundant insect pollinators was assessed by measuring stay time and visitation rate using a digital stopwatch. Stay time was estimated by observing the duration an individual pollinator spends on a flower, whereas visitation rate was calculated by observing the number of flowers visited by a pollinator species in a single minute [31,32]. These observations were recorded three times a day at 08:00, 12:00, and 16:00 h during the flowering period [10].

### 2.4. Impacts of Pollination Treatments on Fruit Set, Yield and Quality

To assess the influence of insect pollinators in determining the yield and physico-chemical properties of jujube fruit, both self-pollination (no insect visit) and cross-pollination (free insect visit) treatments were applied in all three cultivars. For self-pollination, a total of fifty inflorescences were caged in each cultivar using a muslin cloth bag (twelve in each direction, with two directions randomly receiving one extra cage). For cross-pollination data, fifty inflorescences of each jujube cultivar were randomly selected and tagged from every direction (east, west, north, and south). Fruits were harvested from both treatments in all three cultivars and were brought to the laboratory for an analysis of different yield parameters.

#### 2.4.1. Impact of Pollination Treatments on Physical Characteristics and Yield Parameters of Set Fruits

Initially, we counted the total number of fruits per panicle in both treatments (open and caged) using visual observation across all three cultivars. To measure the physical parameters of fruit, we randomly selected 50 fruits from each pollination treatment, both caged and open-pollinated, for each cultivar at two maturity levels. Fruit length and width were measured using a digital vernier calliper. To measure fruit firmness, we used a digital fruit firmness tester (FR-5120, Lutron Electronics Enterprises, Taipei, Taiwan) equipped with an 8 mm tip and mounted on a stand. Fruit and pulp weights were measured using a digital weighing balance.

#### 2.4.2. Impact of Pollination Treatment on Biochemical Characteristics of Set Fruits

To determine the impact of insect pollination on the biochemical characteristics of jujube, four parameters were recorded from the extracted juice of pre-mature and mature fruits harvested from both the caged and open treatments. These parameters included total soluble solids (TSS), titratable acidity (TA), pH, and vitamin C. Abbe’s refractometer was used to determine TSS from the extracted juice of harvested fruit, and the data was recorded as percent TSS. The refractometer apparatus was calibrated with purified water and adjusted to 40 °C. After cleaning the lens of the apparatus with toluene, 2 to 3 drops of jujube juice were deposited on the lens surface, and the reading was recorded. Titratable acidity was determined by diluting 10 mL of clear fruit juice without any suspension from each treatment with distilled water (1:4) and titrating against 0.1 N NaOH in the presence of phenolphthalein as an indicator [33]. Moreover, a digital pH metre was used to calculate the acidity of the fruit juice to take pH readings. The bulb of the pH metre was dipped in 10 mL of jujube juice, and the reading of the metre was noted. The vitamin C (ascorbic acid) content of jujube juice was measured by taking 10 mL of juice in a 100 mL flask, and the rest of the flask was filled with a 0.4% oxalic acid solution. Then, 5 mL of this solution was titrated against 2,6-dichlorophenol indophenol according to the AOAC method till the endpoint [34].

### 2.5. Statistical Analyses

#### 2.5.1. Abundance and Diversity of Flower Visitors of Jujube

We combined the total visit counts for each combination of variety Ayuba (AYB), Desi (DES), Kheri (KHR) and type of insect visitor (bee, fly, wasp, moth, butterfly). Homogeneity of variances was checked using Levene’s test [35], and residual normality was assessed via Shapiro–Wilk test [36]. Since these assumptions were not met, we employed nonparametric tests throughout. Differences among varieties and groups were assessed by Kruskal–Wallis tests [37], and significant group-level effects were followed by Dunn’s multiple-comparison tests [38] with Benjamini–Hochberg adjustment [39]. We report omnibus χ^2^ statistics and adjusted *p*-values; post hoc *p*-values and significance levels are in Table 1, Table 2 and Table 3.

#### 2.5.2. Insect Community Composition Across Varieties

To explore whether the composition of flower-visiting insects differs among the three *Z. mauritiana* varieties, we conducted Non-metric Multidimensional Scaling (NMDS) on the Bray–Curtis dissimilarity matrix of species-level abundances (bootstrapped Wisconsin double–standardised counts). Two-dimensional NMDS converged on a stress of 0.00, indicating an exact representation of pairwise distances in two dimensions. Cultivar centroids in NMDS space were plotted alongside species vectors drawn toward the cultivar(s) with which each insect was most closely associated. Due to having only one community sample per cultivar, formal PERMANOVA was not performed; instead, we assessed biological differences using spatial distances among cultivar centroids in NMDS space.

#### 2.5.3. Visit Duration of Pollinators Among Varieties

We restricted our formal stay-time analysis to the two most abundant visitor groups (bees and flies), both of which had at least six visits per variety, to meet the assumptions of ANOVA. Visit-duration (seconds) was first screened for homogeneity of variances (Levene’s test) and normality of two-way ANOVA residuals (Shapiro–Wilk); both assumptions were met (all Levene’s *p* > 0.05; Shapiro–Wilk *W* = 0.98, *p* = 0.45). We then fitted a two-way ANOVA with the main effects of Variety and Pollinator Type and their interaction:

Visit Duration seconds∼Variety+Pollinator Type+Variety: Pollinator Type

Because the Variety × Pollinator Type interaction was not significant (F_(2, 58)_ = 2.33, *p* = 0.106, we focused on the main effect of Pollinator Group. Pairwise contrasts on Pollinator Group were carried out with Tukey’s HSD, with *p*-values adjusted by the Benjamini–Hochberg procedure. All tests used α = 0.05.

#### 2.5.4. Impact of Pollination Treatments on Physical Characteristics and Yield Parameters of Set Fruits

##### Impact of Pollination Treatments on Fruit Set

Observed fruit-set proportions (No Set/No.Flowers) were first summarised by treatment (Caged vs. Open) and variety (AYB, DES, KHR). Overdispersion was assessed by fitting an intercept-only binomial GLM and computing the Pearson dispersion statistic (ϕ = 1.08), indicating no substantial overdispersion, so a standard binomial model was appropriate. We then fitted the following logit model:

$$logit\;\left(p\right)=\log\frac{p}{1-p}=\;\beta_{0}+\beta_{1}{Pollination}_{open}+\;\beta_{2}{Variety}_{DES}+\;\beta_{3}{Variety}_{KHR}$$

where ${Pollination}_{open}=1$ for open-pollinated and 0 for caged, and Variety factors are compared to the AYB reference. Coefficients were exponentiated to obtain odds ratios (OR) with 95% confidence intervals. We also tested for a pollination × variety interaction in the binomial GLM to assess whether the effect of open pollination varied across varieties, reporting the χ^2^ statistic and *p*-value for the interaction term.

##### Impact of Pollination Treatments on Fruit Firmness

Fruit-firmness data were analysed using a two-way ANOVA to test the effects of Variety (AYB, DES, KHR) and Treatment (Caged vs. Open) on fruit firmness (N). Before analysis, homogeneity of variances was confirmed by Levene’s test (*p* = 0.2341), and the normality of ANOVA residuals was verified by a Shapiro–Wilk test (W = 0.9928, *p* = 0.9785). The model was fitted as follows:

Firmnessijk=μ+αi+βj+(αβ)ij+εijk

where αi is the effect of the *i*th variety, βj the effect of the *j*th treatment; and εijk the residual error. ANOVA table entries (degrees of freedom, sum of squares, mean squares, F-statistics, and *p*-values) are reported. Because the Variety  ×  Treatment interaction was non--significant, we examined main-effect pairwise comparisons of treatment within each variety using estimated marginal means (emmeans) and a compact-letter display.

##### Impact of Pollination Treatments on Fruit Physical Parameters

For each trait (fruit length, fruit width, total fruit weight, and pulp weight), we first summarised the data by Variety (AYB, DES, KHR) and Treatment (Caged vs. Open), reporting mean ± SE. We then fitted separate two-way ANOVAs:

Traitijkl=μ+αi+βj+(αβ)ij+εijkl,

where αi is the effect of the *i*th variety, βj is the effect of the *j*th pollination treatment, and εijkl is the residual error. Homogeneity of variances (Levene’s test) and normality of residuals (Shapiro–Wilk) were acceptable for all traits.

##### Multivariate Structure of Fruit Quality Traits

We used Principal Component Analysis (PCA) to summarise how fruit physical traits, i.e., length, total weight, pulp weight, and firmness, co-vary across cultivars and pollination treatments. We also generated two PCA biplots: one coloured by pollination treatment to highlight treatment effects, and another by cultivar to emphasise varietal differences in trait syndromes.

#### 2.5.5. Impact of Pollination Treatments on Biochemical Characteristics of Set Fruits

Before any inferential testing of the physiological traits (pH, titratable acidity [% TA], total soluble solids [Brix], and vitamin C [mg 100 g^−1^]), we inspected each dataset for normality (Shapiro–Wilk) and equality of variances (Levene’s test); all traits met these assumptions (all *p* > 0.05). We visualised treatment × variety patterns. Because our primary goal was descriptive, i.e., characterising how open vs. caged pollination affects each quality parameter, we did not pursue formal inferential testing or post hoc contrasts.

## 3. Results

### 3.1. Abundance and Diversity of Flower Visitors of Jujube

**Figure 1:**
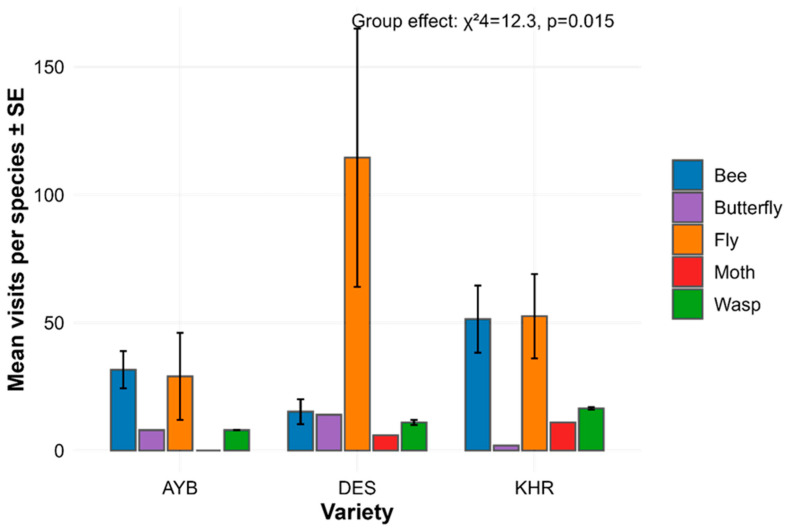
Mean species-level abundance ± SE for each variety and group, annotated with the omnibus group effect.

Total insect counts per species did not differ among the three jujube varieties (Kruskal–Wallis χ^2^_2_ = 0.38, *p* = 0.827; Table 1), indicating overall richness was comparable in AYB, DES, and KHR. In contrast, abundance differed significantly among functional groups (χ^2^_4_ = 12.30, *p* = 0.0153; Table 2). Post hoc Dunn’s tests revealed that bees were significantly more abundant than moths (adjusted *p* = 0.0457) and butterflies (adjusted *p* = 0.0457), whereas comparisons between bees and flies or wasps did not reach significance (all adjusted *p* > 0.28; Table 3). Figure 1 shows that bees dominate mean visits per species in every variety, with flies and wasps intermediate and moths/butterflies rare.

### 3.2. Insect Community Composition Across Varieties

**Figure 2:**
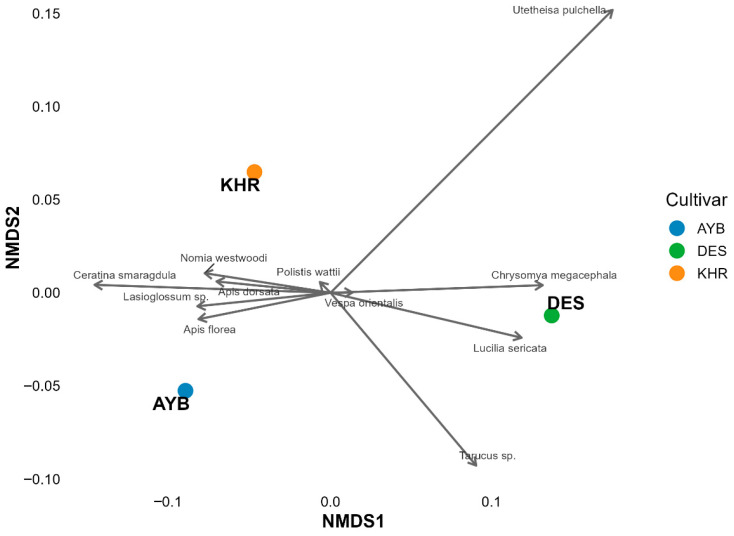
NMDS ordination of insect pollinator communities by cultivar (AYB, DES, KHR) based on Bray–Curtis dissimilarities. Cultivar centroids are shown as large symbols; species labels and vectors point toward the cultivar with which each taxon is most strongly associated. Stress = 0.

The ordination (Figure 2) shows clear separation among cultivars: AYB clusters in the lower-left quadrant, DES in the lower-right, and KHR in the upper-left. Although formal PERMANOVA could not be performed (one community sample per cultivar), the spatial distances among centroids (AYB–DES = 0.231; DES–KHR = 0.200; AYB–KHR = 0.125) support biologically meaningful differences in pollinator assemblages. Bee taxa (*Apis dorsata*, *A. florea*, *Lasioglossum* sp., *Ceratina smaragdula*) load strongly toward AYB, indicating a bee-dominated community. DES is pulled toward Diptera and Lepidoptera, for example, *Chrysomya megacephala* and *Utetheisa pulchella*, while KHR aligns with wasps and solitary bees (e.g., *Polistes wattii*, *Nomia westwoodi*). These patterns suggest cultivar-specific floral traits or phenologies drive differential attraction of insect guilds.

### 3.3. Visit Duration of Pollinators Among Varieties

**Figure 3:**
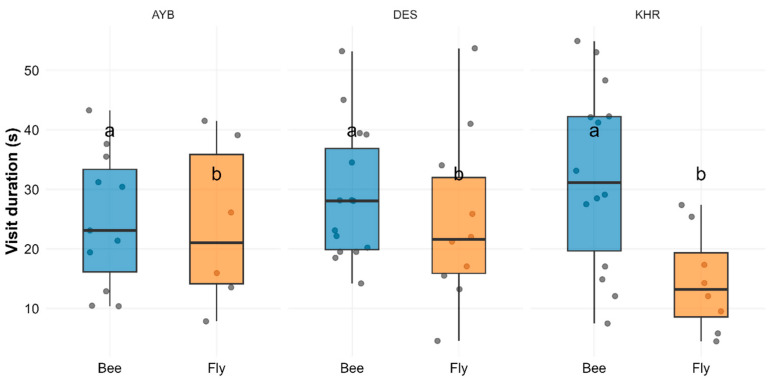
Boxplots with overlaid jitter showing visit duration by pollinator group (bee, fly) for each variety. Error bars represent the interquartile range, and letters above each box (“a” vs. “b”) denote significant group differences (Tukey HSD, *p* < 0.05).

Descriptive statistics (Supplementary Material Table S1a) show mean visit durations of 28.9 ± 2.9 s for bees and 21.9 ± 3.0 s for flies, averaged across varieties. The two-way ANOVA revealed no significant variety × group interaction (F_(2, 58)_ = 2.33, *p* = 0.106) and no main effect of variety (F_(2, 58)_ = 0.21, *p* = 0.811), but a significant main effect of Pollinator Group (F_(1, 58)_ = 5.73, *p* = 0.0199; (Supplementary Material Table S1b). Tukey’s HSD confirmed that bees had significantly longer mean stay times than flies (adjusted *p* = 0.022; (Supplementary Material Table S1c). Figure 3 illustrates these differences across all three varieties, with bees consistently grouping “a” and flies “b.”

### 3.4. Impact of Pollination Treatments on Fruit Set

**Figure 4:**
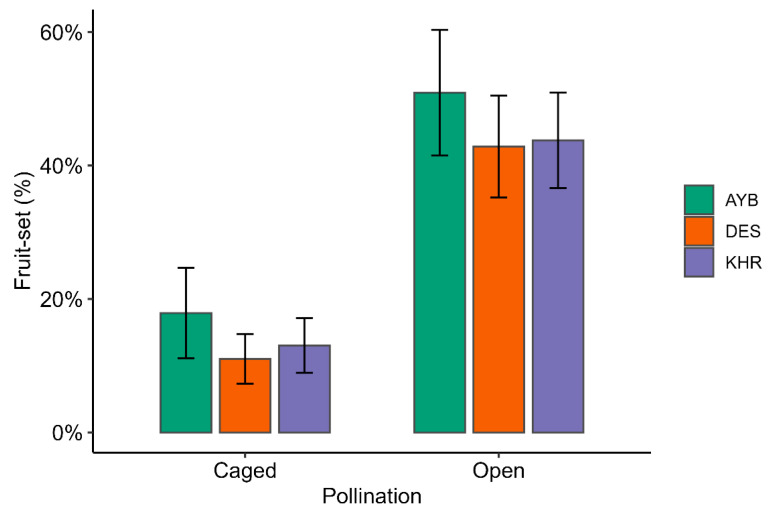
Observed fruit-set proportions by pollination treatment and variety. Bars represent mean proportion fruit-set (%) for caged and open treatments within each variety, with error bars showing 95% confidence intervals.

Across all varieties, open pollination significantly increased the likelihood of fruit-set relative to caged controls: mean fruit-set rose from 12 ± 3% (caged) to 53 ± 5% (open; (Supplementary Material Table S2; Figure 4). In the binomial GLM, open flowers were 5.38 × more likely to set fruit than caged flowers (OR = 5.379; 95% CI 4.133–7.041; z = 12.39, *p* < 0.001; Table 4). Variety DES showed significantly lower set odds compared to AYB (OR = 0.66; 95% CI, 0.47–0.93; *p* = 0.018), while KHR exhibited a similar but nonsignificant trend (OR = 0.72; 95% CI, 0.52–1.02; *p* = 0.065). There was no significant pollination × variety interaction (χ^2^_2_ = 1.48, *p* = 0.48), indicating that the benefit of open pollination was consistent across all three varieties.

### 3.5. Impact of Pollination Treatments on Fruit Firmness

**Figure 5:**
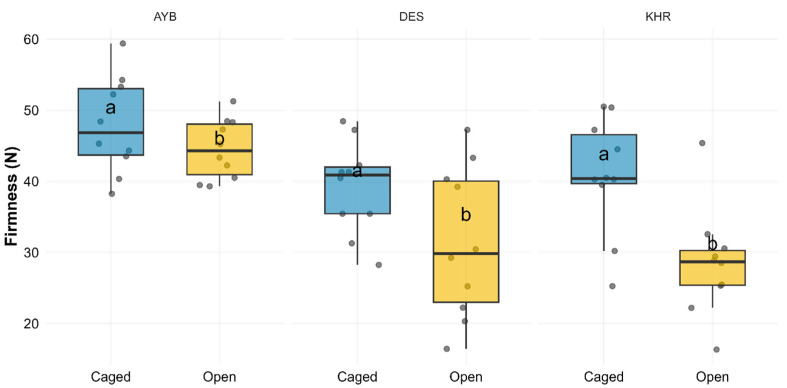
Boxplots of fruit firmness (N) by pollination treatment (Caged vs. Open) faceted by variety (AYB, DES, KHR). Jittered points show individual replicates; letters (a vs. b) denote significant differences between treatments within each variety (Tukey HSD, BH-adjusted *p* < 0.05).

Fruit firmness differed highly significantly among the three varieties (Supplementary Material Table S3a; F_(2, 54)_ = 14.91, *p* < 0.001) and was higher under caged pollination compared to open controls (F_(1, 54)_ = 16.24, *p* < 0.001), while the Variety × Treatment interaction was not significant (F_(2, 54)_ = 1.78, *p* = 0.178). Estimated marginal means showed that caged fruits were significantly firmer than open ones in AYB (47.93 ± 2.13 N vs. 44.54 ± 1.33 N; *p* = 0.012) and in DES (39.15 ± 2.06 N vs. 31.41 ± 3.35 N; *p* = 0.008), but not in KHR (40.87 ± 2.58 N vs. 28.47 ± 2.39 N; *p* = 0.08). These patterns are illustrated in Figure 5, which presents boxplots of individual firmness measurements by treatment and variety, with letters denoting significant within-variety treatment differences. The summary statistics for fruit firmness by variety and pollination treatment are given in Supplementary data Table S3b.

### 3.6. Impact of Pollination Treatments on Fruit Physical Parameters

**Figure 6:**
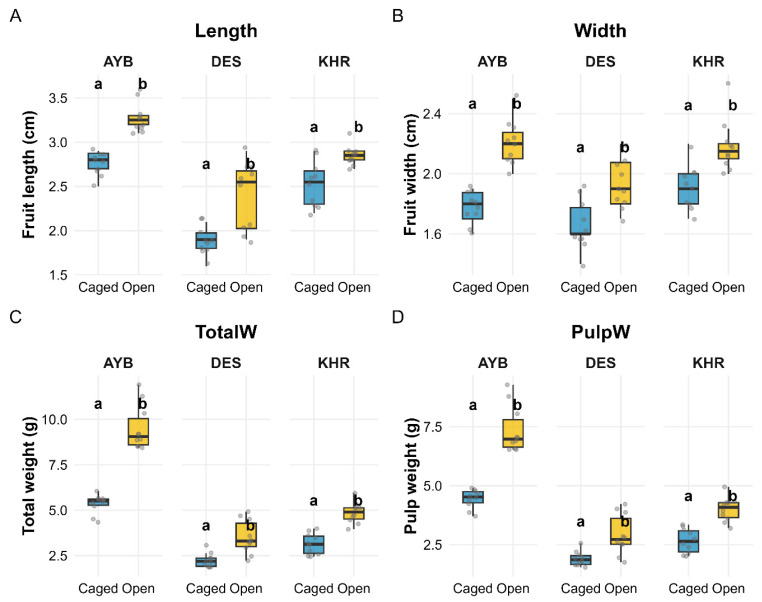
Effects of pollination treatment (Caged vs. Open) on fruit size and weight traits across three varieties (AYB, DES, KHR). Panels (**A**–**D**) show boxplots (jittered points) of (**A**) fruit length, (**B**) fruit width, (**C**) total fruit weight, and (**D**) pulp weight. Within each facet, compact-letter groupings (“a” vs. “b”) denote significant treatment differences (emmeans + BH-adjusted Tukey HSD, *p* < 0.05); letters are placed uniformly just above the taller treatment in each variety.

Summary statistics are presented in (Supplementary Material Table S4a), and ANOVA results are shown in (Supplementary Material Table S4b). Because the length and width interactions were non-significant (both *p* > 0.16), we report their main effects directly. For total weight and pulp weight—both of which had significant Variety × Treatment interactions (*p* < 0.001)—we overlaid compact-letter groupings (emmeans + BH-adjusted Tukey HSD) on Figure 6 to show which treatment differences are significant within each variety.

Fruit size and mass were strongly affected by both variety and pollination treatment. Fruit length increased under open pollination in all three varieties (AYB: 2.76 ± 0.04 cm vs. 3.28 ± 0.05 cm; DES: 1.89 ± 0.05 cm vs. 2.39 ± 0.12 cm; KHR: 2.53 ± 0.08 cm vs. 2.85 ± 0.04 cm). Two-way ANOVA showed highly significant main effects of variety (F_(2, 54)_ = 82.60, *p* < 0.0001) and treatment (F_(1, 54)_ = 62.54, *p* < 0.0001), with no variety × treatment interaction (*p* = 0.29).

Fruit width likewise was greater in open-pollinated fruits (AYB: 1.77 ± 0.04 cm vs. 2.20 ± 0.04 cm; DES: 1.66 ± 0.05 cm vs. 1.93 ± 0.05 cm; KHR: 1.91 ± 0.05 cm vs. 2.18 ± 0.06 cm). Both variety (F_(2, 54)_ = 14.71, *p* < 0.001) and treatment (F_(1, 54)_ = 67.69, *p* < 0.001) effects were highly significant, and their interaction was non-significant (*p* = 0.17).

Total fruit weight showed a significant variety × treatment interaction (F(_2_, _54)_ = 21.10, *p* < 0.001), as well as main effects of variety (F_(2, 54)_ = 194.68, *p* < 0.001) and treatment (F_(1, 54)_ = 148.37, *p* < 0.001). Open pollination increased mean fruit weight by 78% in AYB (5.34 ± 0.17 g → 9.51 ± 0.39 g), 57% in DES (2.24 ± 0.12 g → 3.51 ± 0.29 g), and 55% in KHR (3.14 ± 0.18 g → 4.87 ± 0.18 g).

Pulp weight also exhibited a significant interaction (F_(2, 54)_ = 12.49, *p* < 0.001), with variety (F_(2, 54)_ = 157.46, *p* < 0.001) and treatment (F_(1, 54)_ = 111.40, *p* < 0.001) effects. Under open pollination, pulp weight increased by 66% in AYB (4.44 ± 0.13 g → 7.36 ± 0.31 g), 53% in DES (1.91 ± 0.10 g → 2.92 ± 0.27 g), and 50.38% in KHR (2.66 ± 0.16 g → 4.00 ± 0.16 g). All pairwise treatment comparisons within each variety are shown in Figure 6 by “a” vs. “b” letters.

### 3.7. Impact of Pollination Treatments on Fruit Biochemical Parameters

**Figure 7:**
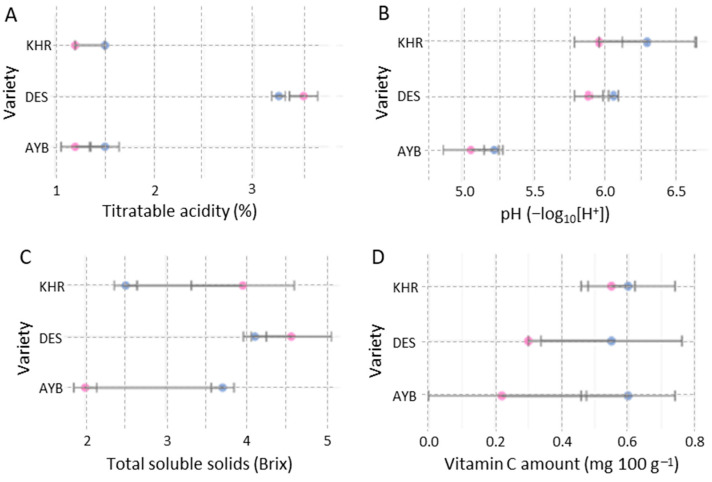
Mean (± SE) of physiological quality traits: titratable acidity (**A**); juice pH (**B**); total soluble solids (**C**); and Vitamin C content (**D**) affected by pollination treatment and variety. In each panel, the y–axis lists the three *Ziziphus* varieties (AYB, DES, KHR). Blue and pink circles denote caged and open treatments, respectively.

Open-pollinated fruits consistently showed higher biochemical regimes than caged fruits across all cultivars (Figure 6). Titratable acidity increased under open pollination, while juice pH declined slightly, indicating greater acidity. Except AYB cultivar, total soluble solids were found to be higher in open fruits, reflecting enhanced sweetness. However, vitamin C content was higher in caged treatments as compared to open-pollinated treatments (Figure 7).

### 3.8. Multivariate Structure of Fruit Quality Traits

**Figure 8:**
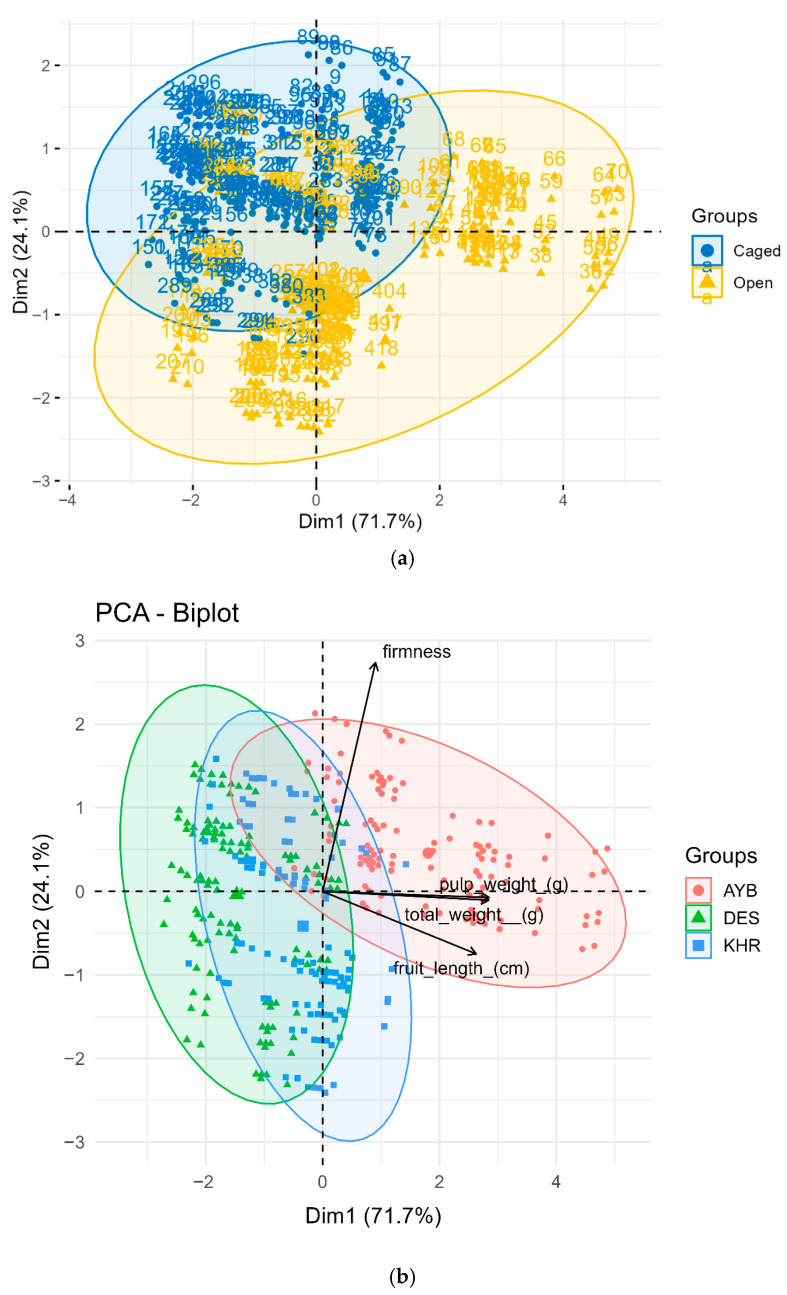
(**a**). Principal component analysis of fruit physical traits, coloured by pollination treatment. Points are individual fruits (blue = caged, gold = open) with 95 % confidence ellipses; vectors show trait loadings on PC1 and PC2. PC1 (71.7 % variance) is driven by pulp weight and total weight, while PC2 (24.1 %) reflects firmness. (**b**). PCA biplot of fruit physical traits across three cultivars. Each point represents one fruit (red = AYB, green = DES, blue = KHR); convex hulls denote 95 % confidence regions. Vectors indicate trait loadings; AYB aligns with large/heavy/firm fruits, DES is intermediate, and KHR clusters near the origin.

Two separate biplots were generated: one colouring points by pollination treatment (Figure 8a) and another by cultivar (Figure 8b). The first two principal components explained 95.8% of the total variance (PC1 = 71.7%, PC2 = 24.1%). In the treatment-based PCA (Figure 8a), open-pollinated fruits (gold triangles) occupy the right-hand tail of PC1, driven by high pulp and total weight loadings (0.99 and 0.99, respectively), whereas caged fruits (blue circles) cluster on the left. PC2 (firmness = 0.95) separates samples vertically, with open fruits slightly lower than caged, reflecting modest treatment differences in firmness. The non-overlapping 95 % confidence ellipses confirm strong multivariate discrimination by pollination mode.

In the cultivar-based biplot (Figure 8b), AYB (red circles) projects far along the positive PC1 axis in the direction of large, heavy, firm fruits. DES (green triangles) falls toward the negative PC1/positive PC2 quadrant—indicating moderate weight but higher firmness—while KHR (blue squares) lies near the origin, signifying average trait values. Vectors for length, total weight, pulp weight, and firmness radiate from the origin, visually defining each cultivar’s “trait syndrome.”

Together, the PCA plots reveal that open pollination engenders a coordinated “quality syndrome” of larger, heavier fruits (high PC1 scores), regardless of cultivar, while cultivar identity independently structures trait space. AYB inherently produces the most robust fruits, DES is intermediate, and KHR the smallest/lightest.

These multivariate analyses reinforce our earlier univariate findings (Objectives 4–5) by demonstrating that both pollination and genotype shape a coherent fruit-quality phenotype across multiple traits.

## 4. Discussion

The pollinator fauna of the jujube orchard in Multan, South Punjab region, was reported for the first time in this study. Jujube flowers attracted a wide range of pollinators, especially from three main insect orders: Hymenoptera, Diptera, and Lepidoptera. Bees were the most abundant functional group (Section 3.1), with previous studies in South Punjab highlighting their efficiency as pollinators across multiple crops [29,30]. Social bees have demonstrated significant pollination efficiency in jujube, as reported in earlier studies [17].

Flowers of the hybrid varieties Ayuba and Kheri were predominantly visited by bees and flies, whereas the non-hybrid variety DES was mainly visited by flies. Various previous studies have also reported varietal preferences of floral visitors in apples [40], tomatoes [41], soybeans [42], and strawberries [42]. These differences in pollinator guilds among the three varieties can be explained by the phenomenon of pollination syndrome, which plays an important role in shaping plant–pollinator interactions [43]. Apart from floral traits, which were similar in all three varieties of jujube, nectar composition is also a dominant feature of pollination syndrome [44]. Sugars and amino acids in nectar provide essential nutrients to nectar feeders [45]. Different pollinator guilds have distinct floral preferences [46], which are driven by the partitioning of nectar resources among insect species depending on the sugar profile of flower nectar [47]. Studies have reported that flowers with higher amounts of sucrose attract bees [48], whereas flies are attracted to nectar rich in hexoses [49]. Flies prefer hexose-rich nectars because they are composed of monosaccharides that could be readily absorbed in their midgut, whereas sucrose needs hydrolysis before being digested. On the other hand, bees prefer sucrose-rich nectar because of its high calorific value [50]. It remains to be determined whether jujube flowers of non-hybrid varieties are rich in monosaccharides and flowers of hybrid varieties produce more sucrose, which could determine the varietal preferences of different pollinator guilds.

In the present study, insect-mediated cross-pollination resulted in an approximately three- to four-fold increase in fruit set proportions under the open pollination treatment compared with the self-pollination treatment, in which pollinator access was restricted. Similar trends have been reported in other *Ziziphus* species; for example, in *Z. jujube*, fruit set under open pollination was 30 times higher than under self-pollination [51], and in *Z. mistol*, insect pollination increased fruit set by 33% [52]. The substantial increase in jujube fruit set through open pollination is mainly due to its self-incompatible flowers, which require cross-pollination. Although jujube flowers are hermaphroditic, there is a temporal separation of the male and female sexual phases, and anther dehiscence occurs before the stigma receptivity [13]. The presence of this strict dichogamy in jujube flowers likely evolved as an adaptive mechanism to minimise the fitness costs of hermaphroditism [53]. In hermaphroditic flowers, self-pollination results in inbreeding depression that compromises reproductive capacity and population survival in plants [54]. For example, in *Z. mauritiana*, it causes early fruit drop [55], and in *Z. jujube*, it leads to the formation of small fruits without viable seeds [56].

In all three jujube varieties, insect-facilitated cross-pollination enhanced all the physical pomological parameters (fruit length, fruit weight, pulp weight, and firmness). Similarly to the current findings, previous studies have also reported improvements in physical parameters, such as fruit length and weight of tomatoes [22], apples, pears [57], litchis [19], and avocados [20] due to insect pollination. The observed increase in fruit length and fruit weight through insect pollination could be due to the enhanced production of the phytohormones auxins and gibberellins. Pollinator-mediated fertilisation of a higher number of ovules by viable pollen results in a higher seed set, which subsequently leads to increased production of growth-related phytohormones [58]. These hormones stimulate both cell division and enlargement in developing ovaries, thereby enhancing fruit size and weight [59]. A higher seed set also promotes the mobilisation of photo-assimilates toward the fruit, which increases the fruit pulp mass [60]. Fruit firmness, which is an important determinant of post-harvest shelf life, also depends on insect pollination [61]. In our study, fruit firmness in all three varieties was lower in open-pollinated fruits, indicating a higher ripening progression commonly associated with enhanced sugar accumulation and consumer acceptability; however, this may also reduce post-harvest storage life [62,63]. The changes in the physical traits of open-pollinated fruits that varied among varieties may have resulted from underlying genotypic differences and from variations in the composition of pollinator guilds [64]. The Apidae (bee) family was found to be the most effective pollinating group among all insects, probably due to its specialised pollen-carrying structures such as branched hairs, scopae, and corbiculae [65]. These structures enable the honey bee to carry a greater pollen load with a higher percentage of viable pollen compared to most of the dipterans [66]. Additionally, bees show higher floral fidelity than do flies, consecutively visiting flowers of the same species in a single foraging trip [67]. This behaviour ensures conspecific pollen transfer with effective pollen deposition, thereby improving fertilisation. This improved fertilisation leads to better fruit development, which is strongly linked with fruit morphology and provides fruits with better shape and dimensions.

It was observed that the foraging behaviour of insect species also had an impact on the physical parameters of fruits. Short and frequent visits from insect pollinators positively influenced fruit yield and fruit quality in all jujube varieties. Shorter visits by pollinators reduce floral resource monopolisation, facilitating access for more pollinators to the same flower, ultimately increasing chances of successful cross-pollination, while frequent visitation compensates for limited pollen deposition during each shorter visit by enabling more consistent and repeated pollen transfer [68]. Furthermore, a high visitation rate by a diverse range of insect species promotes pollen mixing from various genetically different plants, leading to the development of more viable seeds and fruits.

Open pollination by insects significantly enhances biochemical reactions in all three varieties, resulting in fruit with a better taste. Insect-mediated open pollination enhanced the biochemical profile of jujube fruits, producing sweeter, more acidic, and vitamin C-rich fruits compared to caged treatments. Changes in the fruit biochemical profile due to insect pollination have also been reported in previous studies on strawberry [69], litchi [19], sweet cherry [25], and orange [24]. Elevated auxin production in open-pollinated fruits promotes more sugar accumulation by regulating photo-assimilates. Overall, insect pollination significantly improved fruit yield, fruit shape, and taste in all three varieties of jujube, highlighting the importance of insect pollinators. Honey bees proved themselves to be the most effective taxa among all the pollinator guilds in the jujube orchard in terms of abundance and visitation rate, especially *Apis dorsata*. Dipterans also played a significant role in the pollination of one of the jujube varieties, highlighting that, beyond bees, maintaining a diverse pollinator fauna is also necessary to guarantee the reproductive success of a crop. Apart from honey bees, emphasis should be given to the conservation of solitary bees and flies by providing them with year-round foraging resources in the form of conservation strips around orchards and crops [70], along with the use of safer insecticides [71,72]. Modifications in orchard management practices should be made to include insect pollination as a valuable input for yield enhancement [32].

## 5. Conclusions

The current study shows the role of insect pollinators in improving the reproductive success and fruit quality of *Ziziphus mauritiana*. Open pollination increased fruit set three to four times and produced larger, heavier, and sweeter fruits with higher pulp mass and titratable acidity compared to the treatment with no pollination. Cultivar-specific pollinator assemblages, such as bee dominance in the Ayuba cultivar (AYB), fly predominance the in Desi cultivar (DES), and mixed guild activity in Kheri (KHR), show that nectar chemistry and floral rewards influence pollinator attraction and efficiency. Open pollination also reduced fruit firmness, indicating faster ripening and a potential trade-off between sweetness and storage life. Vitamin C content was higher in caged fruits. Overall, these findings highlight the importance of pollination as a vital ecosystem service essential for enhancing jujube yield, fruit quality, and long-term orchard sustainability.

## Figures and Tables

**Table 1:** Kruskal–Wallis test comparing total insect counts per species among the three jujube varieties. χ^2^_2_ = 0.38, *p* = 0.827, indicating no significant difference in overall abundance across AYB, DES, and KHR.

Insect Type	Number of Observations (n)	Test Statistic (H)	Degrees of Freedom (df)	*p*-Value	Test Method
Total	15	0.38	2	0.827	Kruskal–Wallis

**Table 2:** Kruskal–Wallis test comparing total insect counts per species across the five functional groups. χ^2^_4_ = 12.30, *p* = 0.0153, indicating a significant effect of group on abundance.

Insect Type	Number of Observations (n)	Test Statistic (H)	Degrees of Freedom (df)	*p*-Value	Test Method
Total	15	12.3	4	0.0153	Kruskal–Wallis

**Table 3:** Dunn’s pairwise post hoc comparisons (Benjamini–Hochberg adjusted) among insect groups for total counts per species. Bees were significantly more abundant than moths and butterflies (adjusted *p* = 0.0457 for both), with no other pairwise differences reaching significance.

Insect Type	Group 1	Group 2	n1	n2	Statistic	*p*-Value	Adjusted *p*	Significance
Total	Bee	Butterfly	3	3	−2.46	0.013	0.045	*
Total	Bee	Moth	3	3	−2.73	0.006	0.045	*
Total	Fly	Moth	3	3	−2.46	0.013	0.045	*
Total	Butterfly	Fly	3	3	2.19	0.028	0.071	ns
Total	Bee	Wasp	3	3	−1.36	0.170	0.284	ns
Total	Moth	Wasp	3	3	1.36	0.170	0.284	ns
Total	Butterfly	Wasp	3	3	1.09	0.273	0.341	ns
Total	Fly	Wasp	3	3	−1.09	0.273	0.341	ns
Total	Bee	Fly	3	3	−0.27	0.784	0.784	ns
Total	Butterfly	Moth	3	3	−0.27	0.784	0.784	ns

* = significant (adjusted *p* < 0.05); ns = not significant (adjusted *p* ≥ 0.05).

**Table 4:** A binomial generalised linear model assessing the effects of pollination treatment and variety on fruit-set probability. Coefficients are presented as odds ratios (OR) with 95% confidence intervals, along with Wald *z*-statistics and *p*-values. The reference levels are caged pollination and AYB variety.

Term	Estimate of Effect	Standard Error (SE)	Test Statistic (z-Value)	*p* Value	Lower Confidence Interval	Upper Confidence
(Intercept)	0.2024107	0.1596992	−10.00291	<0.001	0.1470836	0.2752055
Pollination (Open)	5.3789266	0.1357845	12.390873	<0.001	4.1333744	7.0408788
Variety (DES)	0.6563619	0.1757562	−2.3956084	0.0184532	0.4652092	0.9270674
Variety (KHR)	0.7249024	0.172213	−1.8681418	0.06467	0.517507	1.0170385

## Data Availability

The original contributions presented in this study are included in the article/Supplementary Materials. Further inquiries can be directed to the corresponding authors.

## Supplementary Materials

The following supporting information can be downloaded at: https://www.mdpi.com/article/10.3390/insects16121183/s1.
