# Supplementary material for: Insect Pollination Enhances Yield and Physicochemical Quality Traits in Three Jujube Cultivars
Source: Insects. 2025 Nov 21;16(12):1183. doi: 10.3390/insects16121183 (PMC12734124; doi:10.3390/insects16121183)
Supplement: Supplementary file 1 [file insects-16-01183-s001.zip › insects-3807702-supplementary.pdf]

Table S1a. Summary of visit durations by pollinator group and variety. Shown are sample size (n), mean (± SE), standard deviation (SD), and standard error (SE) of stay time (seconds) for bees and flies on each variety.

Variety	Pollinator Type	Sample size (n)	Mean Stay time (s)	Standard deviation (SD)	Standard Error (SE)
AYB	Bee	11	25.072	11.364	3.42
AYB	Fly	6	24.026	13.959	5.69
DES	Bee	15	28.882	11.198	2.891
DES	Fly	10	24.837	14.505	4.587
KHR	Bee	14	32.265	15.394	4.114
KHR	Fly	8	14.546	8.4476	2.986

Table S1b. Two-way ANOVA for visit duration (seconds) testing the main effects of variety and pollinator group. Degrees of freedom (Df), sum of squares (SS), mean squares (MS), F-statistics (statistic), and p-values are reported; the variety × group interaction was non-significant.

Variable	df	SS	MS	F-statistic	*P* value
Variety	2	68.736253	34.368127	0.2099236	0.8112593
Pollinator type	1	938.50811	938.50811	5.7324931	0.0199069
Variety x Pollinator type	2	762.3853	381.19265	2.3283594	0.1065001
Residuals	58	9495.6016	163.71727		

Table S1c. Tukey's HSD pairwise comparison of mean visit duration between pollinator groups (Bee vs. Fly), with Benjamini–Hochberg–adjusted p-values indicating significantly higher stay time for bees.

Term	Group 1	Group 2	Null Value	Estimate	Confidence Interval (Low)	Confidence Interval (High)	Adjusted *P*	Significance
Type of Pollinator	Bee	Fly	0	-7.814	-14.48	-1.146	0.022	*

Table S2. Summary of fruit-set by pollination treatment and variety. For each combination of variety (KHR, DES, AYB) and pollination treatment (Caged, Open), the total number of flowers observed, total fruits set, and resulting proportion of fruit-set (± 95 % CI) are shown.

**Variety**	**Pollination type**	**Total Flowers (n)**	**Total Fruit Set (n)**	**Proportion of fruit set**	**Number of plots**
AYB	Caged	123	22	0.1788618	12
AYB	Open	108	55	0.5092593	12
DES	Caged	272	30	0.1102941	20
DES	Open	161	69	0.4285714	20
KHR	Caged	261	34	0.1302682	20
KHR	Open	185	81	0.4378378	20

Table S3a. Two-way ANOVA results for fruit firmness testing the main effects of variety and pollination treatment, and their interaction. Degrees of freedom (Df), sum of squares (SS), mean squares (MS), F-statistics, and *p*-values are reported; the variety × treatment interaction was non-significant.

**Term**	**df**	**Sumsq**	**meansq**	**statistic**	***p* value**
Variety	2	1694.97	847.487	14.9141	< 0.001
Treatment	1	922.768	922.768	16.2389	< 0.001
Variety:Treatment	2	202.798	101.399	1.78442	0.17765
Residuals	54	3068.52	56.8244		

Table S3b. Summary of fruit firmness by variety and pollination treatment. Shown are the number of observations (n), observed mean firmness, standard deviation (SD), and standard error of the mean (SE) for each combination of variety (AYB, DES, KHR) and treatment (caged, open).

**Variety**	**Treatment**	**n**	**Mean**	**SD**	**SE**
AYB	Caged	10	47.93	6.75032	2.13464
AYB	Open	10	44.54	4.2021	1.32882
DES	Caged	10	39.15	6.50291	2.0564
DES	Open	10	31.405	10.5801	3.34572
KHR	Caged	10	40.865	8.15131	2.57767
KHR	Open	10	28.47	7.55326	2.38855

Table S4a. Summary of fruit size and weight traits by variety and pollination treatment. For each trait (Length, Width, TotalW, PulpW), variety (AYB, DES, KHR), and treatment (Caged, Open), shown are the number of fruits (n), mean value, standard deviation (SD), and standard error of the mean (SE).

Trait	Variety	Treatment	N	Mean	SD	SE
Fruit	AYB	Caged	10	2.76	0.13499	0.04269
Length	AYB	Open	10	3.28	0.16193	0.05121
	DES	Caged	10	1.89	0.15239	0.04819
	DES	Open	10	2.39	0.3755	0.11874
	KHR	Caged	10	2.53	0.25408	0.08035
	KHR	Open	10	2.85	0.11785	0.03727
Pulp	AYB	Caged	10	4.442	0.40373	0.12767
Weight	AYB	Open	10	7.358	0.99191	0.31367
	DES	Caged	10	1.907	0.30847	0.09755
	DES	Open	10	2.916	0.8499	0.26876
	KHR	Caged	10	2.66	0.51307	0.16225
	KHR	Open	10	4.003	0.5134	0.16235
Total	AYB	Caged	10	5.34	0.5273	0.16675
Weight	AYB	Open	10	9.512	1.23208	0.38962
	DES	Caged	10	2.239	0.38866	0.1229
	DES	Open	10	3.511	0.92379	0.29213
	KHR	Caged	10	3.142	0.57352	0.18136
	KHR	Open	10	4.865	0.57666	0.18236
Fruit	AYB	Caged	10	1.77	0.11595	0.03667
Width	AYB	Open	10	2.2	0.14142	0.04472
	DES	Caged	10	1.66	0.16465	0.05207
	DES	Open	10	1.93	0.16364	0.05175
	KHR	Caged	10	1.91	0.14491	0.04583
	KHR	Open	10	2.18	0.17512	0.05538

Table S4b. Two-way ANOVA results testing the effects of Variety, Treatment, and their interaction on each fruit trait. For each Trait, the table reports term, degrees of freedom (df), sum of squares (SS), mean squares (MS), F-statistic, and *p*-value.

**Trait**	**term**	**df**	**SS**	**MS**	**F-statistic**	***P* value**
Fruit Length	Variety	2	7.90533	3.95267	82.6022	<0.001
	Treatment	1	2.99267	2.99267	62.5402	<0.001
	Variety: Treatment	2	0.12133	0.06067	1.2678	0.28969
	Residuals	54	2.584	0.04785	NA	NA
Fruit Width	Variety	2	0.68133	0.34067	14.705	<0.001
	Treatment	1	1.56817	1.56817	67.6906	<0.001
	Variety: Treatment	2	0.08533	0.04267	1.84173	0.16836
	Residuals	54	1.251	0.02317	NA	NA
Total Weight	Variety	2	224.657	112.329	194.679	<0.001
	Treatment	1	85.6098	85.6098	148.372	<0.001
	Variety: Treatment	2	24.3517	12.1758	21.1021	<0.001
	Residuals	54	31.1578	0.577	NA	NA
Pulp Weight	Variety	2	130.755	65.3774	157.46	<0.001
	Treatment	1	46.253	46.253	111.4	<0.001
	Variety: Treatment	2	10.3709	5.18545	12.489	<0.001
	Residuals	54	22.4208	0.4152	NA	NA
